# Genetic and Environmental Contributions to Serological Biomarkers of Extracellular Matrix Remodeling in Asthma: A Twin Study

**DOI:** 10.1002/clt2.70089

**Published:** 2025-08-04

**Authors:** Matej Andelic, Vibeke Backer, Kirsten Ohm Kyvik, Signe Holm Nielsen, Simon Francis Thomsen

**Affiliations:** ^1^ Department of Biomedical Sciences University of Copenhagen Copenhagen Denmark; ^2^ Immunoscience Nordic Bioscience Herlev Denmark; ^3^ Department of Otorhinolaryngology, Head and Neck Surgery, and Audiology Rigshospitalet University of Copenhagen Copenhagen Denmark; ^4^ Department of Clinical Medicine University of Copenhagen Copenhagen Denmark; ^5^ Department of Clinical Research Research Unit of Clinical Genetics University of Southern Denmark and Odense University Hospital Odense Denmark; ^6^ Department of Dermato‐Venereology and Wound Healing Centre Bispebjerg Hospital Copenhagen Denmark

**Keywords:** asthma, biomarkers, collagens, immunity, obstructions

## Abstract

**Background:**

Asthma is characterized by airway obstruction driven by chronic inflammation, leading to extracellular matrix (ECM) remodeling. This involves ECM alterations, including increased collagen deposition and elastolysis, resulting in airway wall thickening and irreversible airflow limitation. Despite ECM remodeling's known role in asthma, no reliable tools track these changes, and the genetic and environmental factors driving them remain unclear.

**Objective:**

This study investigated ECM remodeling in asthma by assessing 12 serological neo‐epitopes related to collagen synthesis, degradation, and immune cell activity. Studying monozygotic (MZ) and dizygotic (DZ) twins, we explored genetic and environmental influences on ECM changes.

**Methods:**

The study included 512 individuals from 256 twin pairs (89 MZ, 167 DZ), of which 200 were healthy and 312 had asthma. An exploratory panel of 12 ECM biomarkers reflecting type III and VI collagen formation (PRO‐C3, PRO‐C6), turnover (PRO‐C4), degradation (C3M, C4M, C4Mα3, C6M, C7M), and immune cell activity (VICM, ELP‐3, ELA‐HNE, CC16‐HNE) was measured in serum using ELISA.

**Results:**

Asthma was linked to increased type IV collagen degradation (C4M). Airway obstruction showed decreased PRO‐C6, C4Mα3, C6M, and C7M, while C4M and ELP‐3 were elevated among twins. Classical twin analyses revealed genetic influence on multiple biomarkers, primarily C4Mα3, C7M, CC16‐HNE, and VICM.

**Conclusion:**

This study highlights ECM remodeling's role in asthma and airway obstruction, identifying distinct biomarker profiles. Genetic factors significantly influence ECM changes, suggesting potential genetic predispositions for ECM alterations and offering insights into asthma pathogenesis and future diagnostic and therapeutic strategies.

## 1 Introduction

Asthma is a complex, heterogeneous disease characterized by wheeze, shortness of breath, chest tightness, and cough [1, 2]. A hallmark of asthma is airway obstruction, primarily due to a reduction in airway diameter. This narrowing is driven by chronic inflammation within the airway wall, which initiates a dynamic process involving cell de‐differentiation, migration, differentiation, and maturation. These cellular changes contribute to altered connective tissue deposition and ultimately lead to permanent remodeling of airway structures leading to the overall increase in airway wall thickness [2, 3, 4]. Permanent remodeling of airway structures is driven by changes in the extracellular matrix (ECM), characterized by increased collagen deposition and elastolysis.

The ECM of the lungs is composed of various fibrous proteins, including collagens, elastin, and fibronectin, along with a diverse range of proteoglycans and glycoproteins. This complex matrix provides tensile strength and elasticity, allowing lung tissue to withstand the mechanical forces of expansion and recoil [5]. Beyond its structural role, the ECM binds and regulates the availability of various cytokines and growth factors, facilitating interactions with inflammatory cells and influencing key cellular functions such as development, migration, and proliferation [6, 7, 8]. The lung's ECM is composed of two main components: the basement membrane (BM) and the interstitial matrix. In patients with not well controlled asthma, the BM undergoes significant thickening—an early and defining feature of asthmatic airways [9, 10]. This thickening is associated with an increased number and heightened activity of subepithelial myofibroblasts, which contribute to the deposition of ECM components such as tenascin C, fibronectin, perlecan, versican, decorin and collagens [9, 11, 12].

Given the dynamic ECM changes in asthma, identifying biomarkers reflecting these structural changes in lung tissues is of interest. Here, ECM‐specific biomarkers could contribute to the understanding of the tissue changes, and offer insights for disease monitoring, as well as potentially identify therapeutic targets for managing severe asthma. Recent advancements have led to identification of several biomarkers reflecting these changes. For instance, C4Mα3, a biomarker of type IV collagen alpha‐3 chain degradation by matrix metalloproteinase (MMP) 2, 9 and 12, is elevated in experimental asthma mouse models and shown to depend on mast cell chymase, highlighting its potential as a predictive biomarker for anti‐immunoglobulin E (IgE) therapy [13]. Additionally, elevated levels of C4Mα3 have been observed in both adults and children with asthma, correlating with the severe, exacerbating allergic asthma phenotype. Furthermore, C1M, which reflects MMP‐degraded type I collagen, has emerged as a potential marker for a subtype of neutrophilic asthma associated with severity, obesity, and elevated neutrophil levels [14].

On this background, the aim of this exploratory study was to investigate ECM remodeling in individuals with asthma by assessing serological neo‐epitopes related to collagen synthesis, degradation, and immune cell activity in the systemic circulation. By studying both monozygotic (MZ) and dizygotic (DZ) twins, we elucidated the genetic and environmental influences contributing to these changes, thereby enhancing our understanding of asthma pathology and potential therapeutic targets.

## 2 Materials and Methods

### 2.1 Subjects

This cross‐sectional study of adult twin pairs with at least one twin in each pair reporting a history of asthma was conducted between 2003 and 2005 at the Department of Respiratory Medicine, Bispebjerg Hospital in Copenhagen, Denmark. Detailed methodology of the clinical examination of all participants has been previously described [15]. Clinical asthma was defined as repeated episodes of wheezing, shortness of breath, chest tightness, and/or cough at rest, during physical exertion, or in response to allergens or other unknown triggers, without the presence of a respiratory infection. Airway obstruction was defined as a Tiffeneau‐Pinelli index below 0.8.

Inclusion criteria required participants to complete a full clinical assessment, spirometry, fractional exhaled nitric oxide (FeNO) measurement, serum total IgE testing, and skin prick testing. Exclusion criteria included incomplete clinical data, inability to perform lung function testing, or unclear asthma status following clinical evaluation.

Serum samples for biomarker measurements were obtained from all participants and stored at −80°C until analysis. To study the contribution of genetics and environmental factors on variation in biomarkers of ECM remodeling, our investigation employed the classical twin method that assumes that MZ twins share all their genes, along with their upbringing and early environment, while DZ twins share, on average, only 50% of their segregating genes in addition to their shared upbringing [16].

Informed consent was obtained from all participants before enrollment. The study received approval from the local ethics committee (Protocol no. S‐VF‐20010202) and was conducted in accordance with the Declaration of Helsinki and Danish regulations.

### 2.2 ECM Biomarker Measurements

An exploratory panel of ECM biomarkers reflecting collagen formation, degradation and immune‐cell activity were measured in serum using validated competitive enzyme‐linked immunosorbent assays (ELISAs) utilizing monoclonal antibodies (Nordic Bioscience, Herlev, Denmark). All biomarker measurements were conducted following standard operating procedures to ensure consistency in sample handling and assay execution. The analyses were performed in duplicates, with intra‐ and inter‐assay coefficients of variation maintained below 10% and 15%, respectively. The following biomarkers were measured: Formation of type III collagen (PRO‐C3) [17], type VI collagen (PRO‐C6) [18], MMP‐cleaved type III collagen (C3M) [19], type IV collagen α1 chain (C4M) [20], type IV collagen α3 chain (C4Ma3) [21], type VI collagen (C6M) [22], type VII collagen (C7M) [23], and turnover of type IV collagen 7S domain (PRO‐C4) [24]. Additionally, we assessed levels of MMP‐degraded and citrullinated vimentin (VICM) [25], proteinase 3‐degraded elastin (ELP‐3) [26], elastase‐degraded elastin (ELA‐HNE) [27], and club cell protein 16 (CC16‐HNE) (Unpublished). The mentioned biomarkers indicate the involvement of different ECM compartments and immune cell activity in the tissue: PRO‐C3 and PRO‐C6 for fibrosis, PRO‐C4 for basement membrane turnover, C3M for interstitial matrix degradation, C4M and C4Mα3 for basal lamina disruption, C6M for microfibril degradation, and C7M for epithelial damage. VICM was used to assess macrophage activity, while ELP‐3, ELA‐HNE, and CC16‐HNE reflected neutrophil activity. Moreover, the detection ranges of the ELISA assays, expressed in ng/mL, are as follows: PRO‐C3 (24.20–868.80), PRO‐C4 (457.6–59,416.0), PRO‐C6 (1.40–220.00), C3M (136–10,264), C4M (28.0–3924.2), C4Mα3 (0.63–30.00), C6M (61–14,923), C7M (0.50–136.30), VICM (7.4–4513.5), ELP‐3 (1179 to 120,000), ELA‐HNE (17.5–8400.0), and CC16‐HNE (13.37–1000).

### 2.3 Statistics

Descriptive statistics are presented as mean ± standard deviation (SD) for continuous variables, and as counts and percentages for categorical variables. Biomarker data were natural log‐transformed to approximate normality and stabilize variance for statistical analyses. Separate linear mixed models were used to assess differences in biomarker levels by asthma status and airway obstruction status, adjusting for age, sex, and body mass index (BMI). In each model, log‐transformed biomarker data served as the dependent variable, with either asthma status or airway obstruction status included as a fixed effect. Patient‐specific intercepts were modeled as random effects to account for correlated measurements within twin pairs. For this analysis, means are reported as mean ± SD. For the classical twin analysis, the Pearson's correlation in biomarker levels within MZ and DZ twin pairs was computed. Moreover, Pearson's correlations were calculated for co‐twin discordant MZ and DZ twins' analysis based on their asthma and airway obstruction status, and Fisher's *z*‐test was used to compare the strength of biomarker correlations between MZ and DZ twin pairs. A *p*‐value of less than 0.05 was considered statistically significant for all analyses.

All statistical analyses and graphical representations were performed using R Studio (version 4.4.1 for Windows, R Foundation for Statistical Computing, Vienna, Austria).

## 3 Results

### 3.1 Characteristics of Participants

The sample included 512 individuals corresponding to 256 complete twin pairs: 89 monozygotic (MZ) and 167 dizygotic (DZ) pairs (Table 1). Of these 200 were healthy, non‐asthmatic individuals and 312 were asthmatic individuals, which originated from 16 concordant non‐asthmatic pairs, 168 discordant asthmatic pairs, and 72 concordant asthmatic pairs. Among the participants, 153 individuals were identified as having current airway obstruction based on the Tiffeneau‐Pinelli index. Of all participants the mean age was 35.8 (± 8.2), 41.2% were males, and 5.1% were current smokers.

**TABLE 1:** Demographic, clinical, and biochemical characteristics of the study participants.

	No asthma (*N* = 200)	Asthma (*N* = 312)	Total (*N* = 512)	*p* value
Age (years), mean (± SD)	35.7 (8.5)	35.8 (8.0)	35.8 (8.2)	0.91
MZ twins	53 (26.5%)	125 (40.1%)	178 (34.8%)	< 0.01
Males	87 (43.5%)	124 (39.7%)	211 (41.2%)	0.40
BMI (kg/m2), mean (± SD)	25.0 (4.5)	25.4 (4.6)	25.2 (4.6)	0.32
Current smoking	49 (24.5%)	58 (18.6%)	107 (20.9%)	0.15
Ever smoking	92 (46.0%)	143 (45.8%)	235 (45.9%)	0.97
Current SABA	1 (0.5%)	144 (46.2%)	145 (28.3%)	< 0.01
Current LABA	3 (1.5%)	40 (12.8%)	43 (8.4%)	< 0.01
Current ICS	3 (1.5%)	104 (33.3%)	107 (20.9%)	< 0.01
Any inhaled medication	3 (1.5%)	163 (52.2%)	166 (32.4%)	< 0.01
Rhinitis	71 (35.5%)	223 (71.5%)	294 (57.4%)	< 0.01
BHR	13 (7.0%)	119 (41.3%)	132 (27.8%)	< 0.01
RDR, mean (± SD)	2.6 (5.9)	18.3 (55.6)	12.1 (44.2)	< 0.01
FeNO (ppb), mean (± SD)	18.4 (13.3)	30.6 (34.4)	25.8 (28.7)	< 0.01
FEV_1_ (L)_,_ mean (± SD)	3.6 (0.8)	3.3 (0.8)	3.4 (0.8)	< 0.01
FVC (L), mean (± SD)	4.3 (1.1)	4.0 (1.0)	4.1 (1.1)	0.01
Positive SPT	64 (32.0%)	217 (69.6%)	281 (54.9%)	< 0.01
Serum total IgE (kU/L), mean (± SD)	114.6 (318)	206.2 (544)	170.6 (471)	0.03
ECP (μg/L), mean (± SD)	7.82 (6.86)	7.8 (7.1)	7.81 (6.97)	0.98
Current infection	10 (5.0%)	18 (5.8%)	28 (5.5%)	0.89

*Note:* Except where indicated otherwise, number (% of all participants) is presented.

Abbreviations: BHR, bronchial hyperresponsiveness; BMI, body mass index; ECP, eosinophil cationic protein; FeNO, fractional exhaled nitric oxide; FEV_1_, forced expiratory volume in one second; FVC, forced vital capacity; ICS, inhaled corticosteroid; IgE, immunoglobulin E; LABA, long‐acting Beta‐agonist; MZ, monozygotic; RDR, response‐dose‐ratio; SABA, short‐acting Beta‐agonist; SPT, skin prick test.

### 3.2 Reduced C4M Levels Highlight ECM Remodeling in Asthmatic Individuals

**FIGURE 1:**
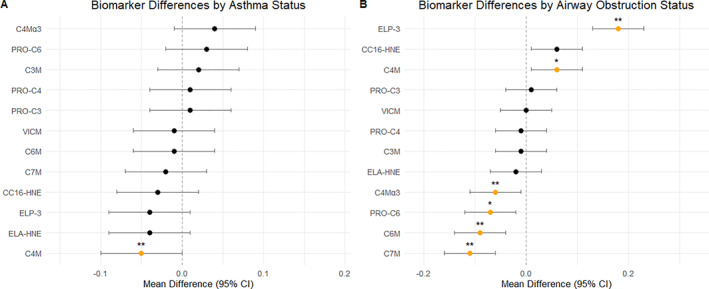
Linear mixed models assessed differences by asthma and airway obstruction status, adjusting for age, sex, and BMI. Fixed effects included asthma or obstruction status, with patient‐specific random intercepts accounting for twin correlations. Data are presented as mean ± SD. Missing data due to high coefficient of variation: PRO‐C3 (4), PRO‐C6 (18), PRO‐C4 (1), C3M (1), C4M (1), C4Mα3 (5), C6M (1), C7M (10), VICM (1), ELP‐3 (1), ELA‐HNE (1), CC16‐HNE (24). Asterisks indicate levels of statistical significance: *p* ≤ 0.05 (*) and *p* ≤ 0.01 (**).

Collagen formation biomarkers showed no statistically significant differences based on asthma status (Figure 1A). However, among the collagen degradation biomarkers, C4M (reflecting type IV collagen α1 chain degradation by MMP12) was significantly reduced in individuals with asthma, with a percentage change of −0.05 (*p* < 0.01). Although crude analysis initially indicated an increase in C4Mα3 in asthmatic siblings, this association became nonsignificant after adjusting for confounding factors (*p* = 0.09). None of the other collagen degradation biomarkers (reflecting types III, VI, and VII) demonstrated significant differences. Similarly, biomarkers reflecting immune cell activity (VICM, ELP‐3, ELA‐HNE, and CC16‐HNE) did not show notable differences between asthmatic and non‐asthmatic individuals (Figure 1A).

### 3.3 Active ECM Remodeling is Linked to Altered Collagen and Elastin Degradation in Individuals With Airway Obstruction

We conducted an analysis to investigate the specific biomarkers differentiating individuals with and without airway obstruction, defined by the FEV1/FVC ratio (Figure 1B).

Among the collagen formation biomarkers, type VI collagen formation was reduced in individuals with airway obstruction, showing a percentage change of −0.07 (*p* = 0.03).

For collagen degradation, the type IV collagen α1 chain biomarker was elevated in individuals with airway obstruction, with a percentage change of 0.06 (*p* = 0.04). Conversely, degradation of the type IV α3 chain was reduced, with a percentage change of −0.06 (*p* < 0.01). Type VI and VII collagens degradation were also reduced in individuals with airway obstruction, with percentage changes of −0.09 (*p* = 0.01) and −0.1 (*p* = 0.01). No significant changes were observed in the degradation of type III collagen between groups.

Elastin degradation, measured by ELP‐3, was significantly elevated in individuals with airway obstruction, with an increase of 0.18 (*p* = 0.01). Other biomarkers related to immune cell activity did not show significant differences between individuals with airway obstruction and their non‐affected counterparts (Figure 1B).

### 3.4 Classical Twin Analysis Indicates Genetic Influence on ECM Biomarker Variation

To perform classical twin analysis, we examined the correlations of biomarker levels in both MZ and DZ twins. We observed significantly higher correlations in MZ twins for C4Mα3, C6M, ELP‐3, and ELA‐HNE, with correlation coefficients of 0.42, 0.24, 0.30, and 0.35, respectively. Additional biomarkers that also exhibited significantly higher correlations in MZ twins compared to DZ twins included C4M, C7M, and CC16‐HNE, contributing to the overall trend of higher correlations in MZ twins (Table 2).

**TABLE 2:** Biomarker correlations in MZ and DZ twins.

Biomarker	MZ correlation	*p* value	DZ correlation	*p* value
PROC3	−0.1	0.17	0.03	0.53
PROC4	0.08	0.27	0.00	0.96
PROC6	0.05	0.53	0.10	0.08
C3M	−0.02	0.77	−0.06	0.31
C4M	−0.18	0.02	−0.13	0.01
C4MA3	0.42	< 0.01	0.15	0.01
C6M	0.24	< 0.01	−0.09	0.08
C7M	0.18	0.02	0.06	0.29
VICM	0.11	0.15	0.02	0.71
ELP3	0.30	< 0.01	0.10	0.06
ELAHNE	0.35	< 0.01	0.02	0.69
CC16HNE	0.19	0.01	−0.16	< 0.01

*Note:* Pearson's correlations were calculated for biomarker levels within MZ and DZ twin pairs.

Abbreviations: DZ, dizygotic; MZ, monozygotic.

**FIGURE 2:**
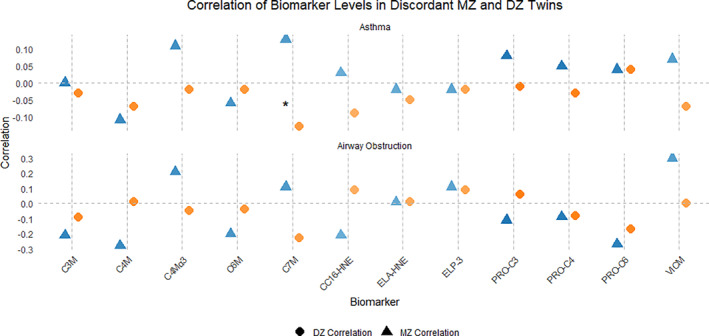
Co‐Twin Control Analysis of Biomarker Correlations in Discordant MZ and DZ Twins. The upper panel presents results based on asthma status, while the bottom panel shows results according to airway obstruction. Pearson's correlations were calculated for discordant MZ and DZ twin pairs, with Fisher's z‐test used to compare correlation strength between twin types. Asterisk (*) represents significant data. DZ, dizygotic; MZ, monozygotic.

Moreover, when analyzing biomarker correlations based on the presence of asthma or airway obstruction in discordant MZ and DZ twins, significant differences in correlation scores were observed for C7M, with a score of 0.13 in MZ twins and −0.12 in DZ twins (*p* = 0.04). Overall, the co‐twin analysis revealed that correlation scores were generally higher in MZ asthma discordant twins for C4Mα3, CC16‐HNE, and VICM. The co‐twin analysis in airway obstruction discordant twin pairs showed a similar trend to that observed in asthma, with higher correlation scores in MZ twins for C4M, C4Mα3, C7M, CC16‐HNE, and VICM (Figure 2) indicating underlying genetic associations between these biomarkers and airway obstruction.

## 4 Discussion

We investigated ECM remodeling in individuals with asthma by analyzing serological biomarkers associated with collagen synthesis, degradation, and immune cell activity. Our findings showed a significant increase in the degradation of type IV collagen α1 chain, C4M, in subjects with asthma. Additionally, the analysis of ECM biomarkers in individuals with current airway obstruction showed significant decrease in PRO‐C6, C4Mα3, C6M and C7M, while C4M and ELP‐3 were significantly elevated in those patients. Moreover, the classical twin analysis revealed genetic propensity for multiple biomarkers, primarily C4Mα3, C6M, C7M, ELP‐3 and ELA‐HNE, in patients with asthma. Additionally, the co‐twin control analysis of discordant twin pairs further supported a genetic association between asthma and/or airway obstruction and C4Mα3, C7M, CC16‐HNE and VICM levels.

Current biomarkers of asthma are primarily derived from induced sputum, blood, exhaled gases, and bronchoscopic samples [28]. Although these biomarkers effectively capture inflammatory processes, they do not provide insights into the structural remodeling of lung tissue—an important component of asthma pathology. Despite extensive research aimed at elucidating the role of airway remodeling in asthma, the absence of clinically validated and accessible biomarkers for assessing this remodeling remains challenging [29]. Moreover, they could facilitate the identification of novel therapeutic targets, thereby offering promising avenues for the management of severe asthma.

Collagen, the most abundant protein in the lungs, undergoes alterations in various lung diseases, including asthma. Its distinct properties—high tensile strength and low elasticity—are vital for preserving lung architecture, with types I, III, IV, V, VII, and XI being the most prominent contributors [30, 31]. Among these, type IV collagen is particularly important in the BM, where it forms a structural network with laminin that supports tissue organization and cellular functions [32, 33]. This collagen subtype is composed of three heterotrimers derived from six distinct type IV collagen chains (α1–α6) [33], where α3 has been shown relevant for chronic asthma [34]. In asthma, a marked reduction in type IV collagen contributes to airway remodeling and dysfunction, by overproduction of mucin 5AC, a protein excessively secreted in the respiratory tracts of asthmatic patients, which exacerbates mucus hypersecretion [35, 36]. Our data indicate that the degradation of the type IV collagen α1 chain is significantly decreased in asthmatic patients, while it is significantly increased in those with airway obstructions. These opposing trends may reflect the chronic consequences of airway remodeling in asthma compared to the active remodeling processes occurring in patients with airway obstruction. One possible explanation for this divergence lies in the differences in the sites and stages of airway remodeling, where both large and small airways are remodeled, and potentially contributing to the opposite findings observed in the two patient groups [37]. Alternatively, this difference could be attributed to the distinct ECM remodeling processes driven by chronic inflammation in asthma versus the acute remodeling and inflammation occurring in obstructed airways [38].

The non‐collagenous domain‐1 of the alpha‐3 chain of type IV collagen (COL4A3), which includes the bioactive fragment tumstatin, is significantly reduced in the lung tissue of asthmatic patients [39, 40]. This reduction is linked to heightened inflammation and angiogenesis, key processes in asthma pathogenesis [41]. In our study, we observed a significant increase in the degradation of the type IV collagen alpha‐3 chain, but this result lost statistical significance after correcting for confounding factors such as age, sex and BMI. However, a trend toward increased degradation remained evident. Interestingly, we also noted a decrease in the same biomarker in patients with airway obstruction, indicating that COL4A3 degradation may play distinct roles in different pathological contexts. COL4A3 may function differently in the chronic inflammatory tissue of asthma compared to the active airway remodeling occurring in patients with obstructive pulmonary disease.

Elastic fibers, which are composed of elastin, are essential for providing the lungs with both resilience and flexibility, as they maintain the expansibility of the alveolar walls, enabling efficient gas exchange and lung compliance [42]. When elastin degrades, it generates fragments, some of which are bioactive and known as elastokines. These elastokines serve various functions, including acting as chemoattractants and promoting lymphocyte proliferation and angiogenesis [43]. Elastokines are implicated in several pathologies, including asthma, chronic obstructive pulmonary disease, cystic fibrosis, and cancer [8, 43, 44].

In our dataset, we observed an increase in ELP‐3 levels, while ELA‐HNE levels remained unchanged, despite both biomarkers reflecting the degradation of elastin. The key difference between these two biomarkers lies in their association with distinct proteases: ELP‐3 is indicative of proteinase‐3 (PR3) activity, while ELA‐HNE reflects the activity of human neutrophil elastase (NE) [45]. A potential reason for the lack of significant elevation in ELA‐HNE, compared to ELP‐3, could be related to the differential release of PR3 and NE upon neutrophil degranulation. In vitro studies have shown that migrating neutrophils release more PR3 than NE from their azurophil granules. Furthermore, some of the PR3 becomes membrane‐bound and is more resistant to inhibition than its counterpart, NE, suggesting that free PR3 has a broader range of activity than NE. This aligns with the hypothesis that the majority of pathological changes in the lungs attributed to NE may, in fact, be driven by PR3 [46, 47].

Furthermore, ELP‐3 levels showed three times higher correlation in MZ twins than in DZ twins, suggesting a genetic predisposition to increased PR3 activity during lung remodeling. We hypothesize that tissue damage occurs when the activity of PR3 surpasses the capacity of protective anti‐proteinases, such as α‐1 antitrypsin, leading to excessive lung tissue degradation [48]. α‐1 antitrypsin deficiency, which is a genetic cause of emphysema and chronic bronchitis in approximately 30% of patients, could explain the results seen in our study [49, 50].

A key strength of this study is its ability to distinguish between chronic inflammation and its outcomes, as well as active lung remodeling in patients. Our findings revealed that the biomarker reflecting type IV collagen α1 chain degradation is decreased in patients with asthma but elevated in those undergoing active ECM remodeling. Another significant aspect of this study is the comprehensive analysis of a panel of different ECM components. The results demonstrated a notable decrease in C4Mα3, C6M, and C7M levels in patients with airway obstructions. This reduction suggests a lack of degradation of these molecules, leading to their accumulation in tissue, which contributes to respiratory tissue stiffness and an asthma‐like phenotype. Conversely, increased levels of C4M and ELP‐3 in patients with airway obstruction highlight the role of type IV collagen and elastin degradation, primarily driven by PR3 activity rather than NE.

To our knowledge, no previous study has examined the structural changes of ECM associated with asthma and/or airway obstructions in twins to discern the influence of genetics versus environment. By utilizing both MZ and DZ twins, we determined whether the observed changes in the ECM biomarkers are attributable to genetic predisposition or environmental influences. The classical twin analysis revealed a genetic influence on multiple biomarkers, primarily C4Mα3, C6M, C7M, ELP‐3, and ELA‐HNE, in patients with lower airways disease. The co‐twin control analysis further supported these findings, reinforcing the role of genetic factors in the regulation of these biomarkers. These results suggest that the significant changes in C4M, C4Mα3, C6M, C7M, and ELP‐3 observed in patients with asthma and airway obstruction are primarily driven by genetic factors rather than environmental ones, contributing to ECM remodeling alterations. It is important to note that detailed data on specific environmental exposures—such as residential conditions, occupational settings, or air quality—were not available, which limits the ability to directly assess their potential influence on these biomarker levels. Future studies should further investigate type IV, VI, and VII collagens, as well as elastin, to better understand the underlying tissue processes. In particular, exploring whether these changes result from disrupted tissue MMP activity or imbalances in tissue inhibitors of MMPs could help develop more efficient therapeutic strategies to improve disease management.

One limitation of studies involving twins is whether the non‐asthmatic twin truly represents the general healthy population or if their characteristics fall somewhere between healthy individuals and asthmatics due to shared genetic and environmental factors with their asthmatic twin [51]. For this purpose, future studies should compare non‐asthmatic twins not only to their asthmatic counterparts but also to a control group of unrelated healthy individuals from the general population. Additionally, the lack of airway‐specific samples such as BALF, bronchial brushings, or biopsies limits our ability to directly assess local airway remodeling and validate serum biomarker findings at the tissue level. Moreover, the absence of comprehensive data on comorbidities unrelated to asthma, such as metabolic or cardiovascular conditions, may have introduced unaccounted variability in systemic biomarker levels. Future studies should incorporate clinical samples, but also a more comprehensive clinical assessment to systematically capture a broader range of comorbid conditions.

A future direction is to measure ECM biomarkers in a longitudinal cohort of asthma patients. This approach would allow determination of whether these biomarkers are associated with disease progression, asthma exacerbations, and the impact of various treatments. None of the patients in the present study were receiving biological therapy, it would be particularly interesting in future investigations to assess ECM biomarker profiles in patients undergoing such treatment. Such data would not only validate the role of ECM biomarkers in monitoring disease dynamics but also provide insights into their utility in predicting clinical outcomes. This would enhance our ability to monitor the underlying pathological processes in asthma, offering a more targeted approach to disease management and therapy optimization.

## Author Contributions

Conceptualization, data curation, formal analysis, methodology, writing – original draft, J.W.; validation, supervision, writing – review and editing, project administration, Q.Y.; investigation, software, A.N. All authors have read and agreed to the published version of the manuscript.

## Conflicts of Interest

The authors declare no conflicts of interest.

## Data Availability

The data that support the findings of this study are available on request from the corresponding author. The data are not publicly available due to privacy or ethical restrictions.
